# Fish and Robots Swimming Together in a Water Tunnel: Robot Color and Tail-Beat Frequency Influence Fish Behavior

**DOI:** 10.1371/journal.pone.0077589

**Published:** 2013-10-25

**Authors:** Giovanni Polverino, Paul Phamduy, Maurizio Porfiri

**Affiliations:** Department of Mechanical and Aerospace Engineering, Polytechnic Institute of New York University, Brooklyn, New York, United States of America; University of Sheffield, United Kingdom

## Abstract

The possibility of integrating bioinspired robots in groups of live social animals may constitute a valuable tool to study the basis of social behavior and uncover the fundamental determinants of animal functions and dysfunctions. In this study, we investigate the interactions between individual golden shiners (*Notemigonus crysoleucas*) and robotic fish swimming together in a water tunnel at constant flow velocity. The robotic fish is designed to mimic its live counterpart in the aspect ratio, body shape, dimension, and locomotory pattern. Fish positional preference with respect to the robot is experimentally analyzed as the robot's color pattern and tail-beat frequency are varied. Behavioral observations are corroborated by particle image velocimetry studies aimed at investigating the flow structure behind the robotic fish. Experimental results show that the time spent by golden shiners in the vicinity of the bioinspired robotic fish is the highest when the robot mimics their natural color pattern and beats its tail at the same frequency. In these conditions, fish tend to swim at the same depth of the robotic fish, where the wake from the robotic fish is stronger and hydrodynamic return is most likely to be effective.

## Introduction

Thousands of fish species are known to aggregate at some stage of their life cycle in organized social groups commonly referred to as “shoals” [1]–[3]. Living in shoals allows fish to reduce the risk of predation [4]–[7] and the energetic costs of their motion [8]–[11]. The coordinated phenomenon of “schooling” is the macroscopic result of a complex transmission of signals within the shoal, in which fish tend to maintain uniform polarization and cohesion in nearly crystallized swimming formations [1], [12].

Such collective behavior is mediated by the integrated physiological system of muscles [13], organs, and senses that has evolved at the individual level as a valid alternative to living solitarily [1], [14], [15]. While it is generally accepted that several sensory cues are utilized by schooling fish to perceive their environment [16] and interact with their conspecifics [17], the quantification of the relative contribution of such cues is yet to be fully understood for several species [9], [15], [18]–[22].

In this context, the use of live stimuli in laboratory experiments only permits minimal flexibility for controlling and dissecting specific behavioral responses. Moreover, natural physiological fluctuations in such stimuli can introduce errors due to the inconsistency of the variables measured [23]. Robotics has been recently proposed as a viable means for enabling hypothesis-driven research in animal behavior, whereby robotic devices can be integrated into animal systems to serve as fully controllable and consistent experimental tools [24]–[26].

In this vein, robots with varying degree of biomimicry have been utilized to influence the behavior of several animal species across an ample set of experimental paradigms tailored to emphasize, and possibly dissect, select biological cues. Visual signaling from biologically-inspired robots has been used to investigate the behavior of birds [27]–[31], dogs [32], lizards [33], fish [22], [34]–[42], and rats [43]; salient chemical cues have been implemented on a miniature mobile robot to investigate social behavior of cockroaches [44]; audio feedback has been integrated in a model of a robotic squirrel to influence squirrels' behavior [45]; pulsing air currents created by robotic honeybees have been used to investigate honeybees' dance [46]; and hydrodynamic cues from a swimming robotic fish have been considered in [20] to modulate fish behavior in a water tunnel. While these efforts have demonstrated the feasibility of using robotics to influence animal behavior, dissecting and quantifying sensory cues in social animals are still untapped research questions. A particularly relevant area entails the analysis of the interplay between visual and flow cues in social freshwater fish.

A variety of phenotypic characteristics observed in freshwater fish species are recognized as important factors in eliciting social interactions between conspecifics [18], [47]–[50]. Fish species used in laboratory studies, such as zebrafish, sticklebacks, and mosquitofish, respond to changes in stripe and color patterns of their conspecifics, and these features have been associated to their shoaling preferences, mating choices, and social ranks, respectively [18], [48], [49], [51]. The same features have been found to be determinants of attraction toward a robotic fish when its morphophysiological and locomotory features have been systematically varied in a series of preference tests [35]–[37]. Specifically, in this series of works it is demonstrated that the behavioral response of zebrafish individuals and small shoals varies as the aspect ratio, color pattern, and tail-beat frequency of a robotic fish is changed. Moreover, the attraction is maximized when the robotic fish most closely replicates its animal counterpart in its color, stripes, and aspect ratio.

Fish swimming pattern is also a critical factor in shaping collective behavior [10], [52]–[55]. Laboratory experiments have demonstrated that fish can be repelled by chaotic and widely fluctuating flow conditions, while being attracted by vortical structures in predictable flows, from which they can harness energy [52], [56]. In [20], golden shiners swam in a water tunnel with a robotic fish whose tail-beat was systematically varied along with the flow speed. While findings in [20] have contributed to validating the hypothesis that the hydrodynamic return offered by a robotic fish is a determinant for robotic fish's attractiveness to live fish, the robot used therein was considerably larger than live fish. The unmatched size between live and robotic fish in [20] may act as a confound for elucidating the role of flow cues produced by fish locomotion on collective behavior. Indeed, fish social behavior is generally dependent on the size-class [57], [58]. Specifically, shoaling in golden shiners is found to be dependent on the size of the neighbors in [19], whereby individuals of the same size-class are preferred shoaling partners.

Controlled animal replicas that incorporate several biologically relevant features from the target species, such as phenotypic cues and locomotion patterns [59], can yield further insight into the social behavior of fish. In this work, we employ two prototypes of robotic fish, whose engineering design was bioinspired to mimic the aspect ratio, body shape, size, and species-specific locomotion pattern observed in live golden shiner. The two prototypes differ in their color pattern that was varied to resemble either the typical pigmentation of their live counterpart or to offer an unnatural color phenotype. The objective of this study is to identify the determinants of attraction that regulate the collective behavior in social fish species when swimming together in a water tunnel. By using a reliable, consistent, and remotely controlled robotic platform, we test the hypothesis that, at a constant swimming velocity, a bioinspired robotic fish is able to elicit attraction in a live fish as a consequence of the visual and flow cues it offers. Specifically, the following predictions are expected to be met: i) fish attraction toward the robotic fish should vary as the visual cues offered by the robotic fish are varied, in agreement with similar observations for zebrafish in [35]–[37]; ii) fish attraction should vary as a function of the robotic fish tail-beat frequency, as suggested in [60] and observed in [20]; and iii) the highest attraction should be reached when both visual and flow cues from the live fish are simultaneously integrated in the robotic fish prototype.

## Materials and Methods

The experiment described in this work was approved by the Polytechnic Institute of New York University (NYU-Poly) Animal Welfare Oversight Committee AWOC-2012-102. Both the housing and the experimental procedure were designed to minimize stress in the animals.

### Bioinspired robotic fish

**Figure 1:**
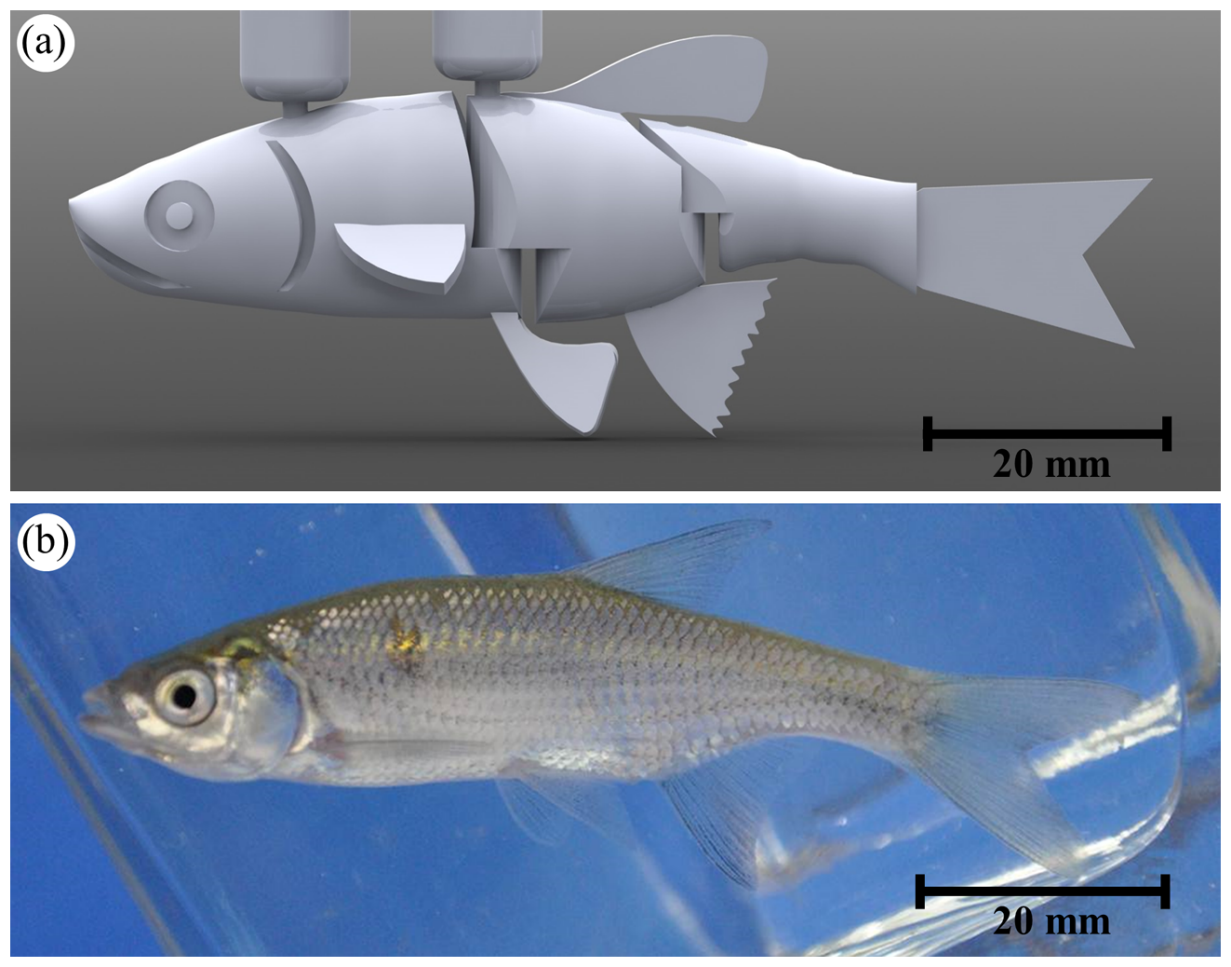
Illustration of the bioinspired robotic fish and golden shiner: (a) computer-aided design of the bioinspired robotic fish connected to the two transparent Plexiglas rods and (b) picture of an experimental golden shiner. (Online version in color).

The robotic fish shown in Figure 1 was used in this experiment. The robotic design included visible fish anatomy, such as a dorsal fin, two pectoral fins, two pelvic fins, an anal fin, and a caudal fin. The robotic fish was composed of three rigid links, interconnected through independent hinges, and a passive silicone caudal fin. The rigid links were designed in SolidWorks and made of solid-packing ABS material printed from a rapid prototyping machine (Stratasys, Dimension SST, USA). The total length was equal to 8.5 cm, the height was 1.8 cm, and the width was 1.0 cm.

Upon completion of the rapid prototyping process, the ABS material was a matte white color. The surface of the robot was painted with non-toxic pigments. One of the two robotic fish was painted to mimic the natural golden shiners' color pattern while another was painted red. The bioinspired colored robotic fish prototype (referred to as “Gray robot”) was painted with a light gray color, and the fins were shaded gray while a black line was drawn along its longitudinal length. The same procedure was used for the red-colored prototype (referred as “Red robot”) whose base color was changed from gray to red. A consistent paint brand (Krylon Products Group, Cleveland, OH) with similar chemical composition was selected to color the two robots and reduce possible confounds associated with olfactory cues. The silicone tail was made using a 0.5 mm deep flat silicone mold and was cut to the required size. Pictures of the prototypes are presented in Figure S1 of Material S1.

In each experiment, a robotic fish was anchored in the water tunnel and was connected to an external servomotor to control its tail-beat frequency. To produce the requisite biomimetic locomotion in the miniature prototype, a waterproof servomotor (GWS Pico + F BB, Grand Wing Servo-Tech, Taiwan) was hosted on a separate platform placed on top of the water tunnel and connected through a transparent Plexiglas rod (8.6 mm diameter) to the most forefront passive joint to regulate the angle between the head and the tail of the robot. Notably, the stationary nature of the robotic fish is based on hardware constraints for mimicking golden shiners' swimming, while maintaining a comparable size and morphology. A second identical rod was used to clamp the head of the robotic fish to the external platform to maintain the first link of the robot parallel to the water flow, see Figure 1a. The robot tail-beat frequency and tail-beat amplitude were controlled by a microcontroller (Arduino Uno, Arduino, Italy) that commanded sinusoidal motions to the servomotor.

### Animals

Golden shiners, *Notemigonus crysoleucas* (length, 7.8±0.5 cm; mass, 5.5±0.3 g; mean±s.d.) were obtained from the Wildlife Conservation Society's Department of Herpetology at the Bronx Zoo (2300 Southern Boulevard Bronx, NY 10460, USA) in June, July, and August 2012 (Figure 1b). Upon their arrival at the laboratory, the fish were transferred to a holding tank with rectangular cross section (0.5 m^2^) with re-circulating, filtered natural freshwater following the procedure in [20]. The fish were acclimated in the Dynamical Systems Laboratory at the NYU-Poly for a minimum of 24 hours before the beginning of the experiments. Golden shiners were kept at constant temperature (23±0.3°C) and the illumination was provided by full spectrum fluorescent lights for ten hours each day, according to circadian rhythm of this species [61]. Animals were fed daily with commercial flake food (tropical fish flakes formula specifically prepared by Petland Discount, Brooklyn) for cyprinid species after the conclusion of the daily experimental session. All fish used in this study were experimentally naïve. Specifically, each tested animal was isolated from the holding tank after the experimental trial to assure that the same individual was not tested multiple times.

### Experimental setup and protocol

**Figure 2:**
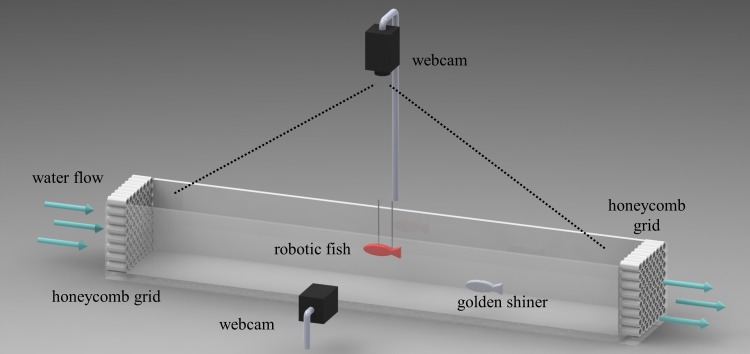
Schematic of the experimental setup (behavioral experiment): the bioinspired Red robot and an individual golden shiner swimming in a section of a Blazka-type water tunnel delimited by two plastic honeycombs. Two webcams are placed above and laterally of the tunnel to record animal activity from two different perspectives. (Online version in color).

Experiments were conducted in a Blazka-type water tunnel [20]. As shown in Figure 2, the observation area was focused on a section of the water tunnel with length 100 cm, width 14 cm, and height 14 cm. Two plastic honeycomb grids were placed on the two opposite sides of the experimental region to limit the observation section while promoting rectilinear flow and uniform velocity profiles, see also [20]. The water flow was induced by an electric pump. The rotational speed of the pump was adjusted to achieve the desired water velocity. The bioinspired robotic fish was tethered to the middle of the working section of the water tunnel with the two support rods, see Figure 1a and Figure 2. Specifically, the center of mass of the robotic fish was placed in the middle of the water column, that is, seven cm from both the water surface and the bottom of the water tunnel, and it was positioned 50 cm from both left and right honeycombs. The center of mass of the robotic fish corresponded to the link from which the input from the external servomotor was transmitted to the robot through the rotation of the Plexiglas rod.

**Figure 3:**
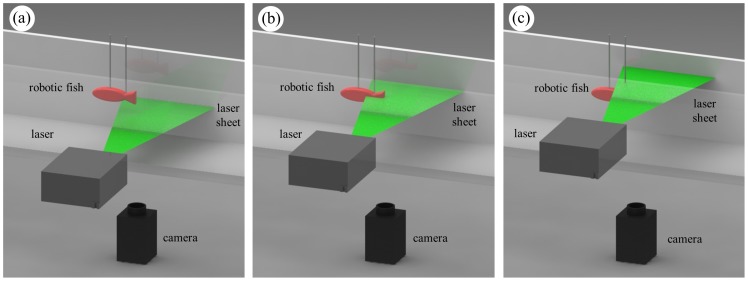
Schematic of the experimental setup (hydrodynamics experiment): the bioinspired Red robot swimming in a section of a Blazka-type water tunnel delimited by two plastic honeycombs (not pictured). A laser sheet oriented in the horizontal (*x*, *y*) perspective illuminates the seeded water in correspondence of the center of the bottom (a), middle (b), and top (c) compartments of the focal region, respectively. A camera placed below the tunnel films the area illuminated by the laser. (Online version in color).

Following standard practice [20], [62], [63], particle image velocimetry (PIV) was implemented in the hydrodynamics experiment of this study to perform instantaneous flow measurements in the wake of the robotic fish, see Figure 3. The water was seeded with silver-coated hollow glass spheres (14 µm diameter, Potters Industries Inc., Carlstadt, NJ, USA) to track the flow. The water tunnel was illuminated by a Solo PIV Nd:YAG Laser (New Wave Research Inc., Fremont, CA, USA) to visualize the particles. The laser was aimed to the side of the robotic fish so that its perspective was orthogonal to the camera axis underneath the water tunnel. Flow images were captured by the MegaPlus Camera ES 1.0 (RedLake MASD Inc., San Diego, CA). The Dantec Dynamics FlowMap 1500 track system (Dantec Dynamics Inc., Denmark) was used to synchronize the laser pulses and camera image captures. Sequential pairs of images (2.5 ms apart in time) were recorded and cross-correlated to study the flow physics in the illumination plane, which was parametrically varied along the depth of the water tunnel, see Figure 3.

In a set of behavioral measurements, fish were observed as they swam together with the robotic fish prototypes. Two webcams (Webcam Pro 9000, Logitech International, Newwark, CA), interfacing with a computer via USB, were synchronized and used to record the fish position in two-dimensional perspectives (Top and Side view) with respect to the robotic fish, see Figure 2. Specifically, an executable script was written in Bash scripting language (GNU operating system, Free Software Foundation, Boston, Massachusetts) to simultaneously collect the video stream from the two webcams. The two webcams filmed the experimental trials at 30 frames per second with a resolution of two megapixels. All frames were used in the behavioral analysis. One of the two webcams was placed above the working section to record the Top view of the water tunnel, and the other was placed laterally with respect to the working section to record the Side view of the water tunnel. The videos were then processed using the software Observer 2.0 (The Observer 2.0, Noldus, Wageningen, The Netherlands) to discriminate for the fish position in each perspective.

Four different experimental conditions were performed on each robotic prototype, that is, the Gray robot and Red robot, as part of the behavioral study. Specifically, the tail-beat frequency of the robotic fish was varied from 0 Hz to 2 Hz, 3 Hz, and 4 Hz. Every condition was repeated six times using different fish for a total number of 48 subjects. For each trial, a single golden shiner was transferred into the water tunnel for the experimental measurements. Following the protocol in [20], the velocity of the water tunnel was initially set to zero while the tail-beat frequency of the robotic fish was set to the specific value. After two minutes, the water velocity was increased from zero to half of the final value, that is, five cm/s. After another 30 minutes the water velocity was further increased to reach its final value, that is, ten cm/s, and it was maintained for 30 minutes before performing a five minutes long experimental trial. Each experiment consisted of a two minutes session with no water flow induced, 30 minutes session with five cm/s of water velocity, 30 minutes session with ten cm/s of water velocity followed by a five minutes experimental trial with ten cm/s of water velocity.

The flow physics studied in the hydrodynamics experiment through PIV was correlated with the results from the behavioral experiment to aid the understanding of fish spatial preference in response to the flow cues. The PIV analyses were performed in multiple regions of the test tank, consistently with the behavioral experiment. In addition, PIV analyses were performed for all the tail-beat frequencies used in the behavioral study in each of the focal compartments. The hydrodynamics and behavioral experiments were not carried out together to avoid possible biasing and/or harmful effects to the fish by the pulsing laser. In all the experimental trials conducted in this study, the water velocity was maintained at ten cm/s (measured using PIV).

### Hydrodynamics and behavioral experiments

The bioinspired robotic fish used in this experiment was designed to mimic the swimming motion of live golden shiners. Biomimicry elements in the experiment were incorporated into the design phase and also assessed from a hydrodynamic standpoint. Structurally, the design of the robotic fish included visible fish appendages with the caudal fin that was cut out from silicone to further emulate the propulsion system observed in its live counterpart. Fish appendage dimensions are presented in Table S1 of Material S1. To assess the degree of biomimicry in the robotic fish design, the undulations of the robotic fish were compared with classical models of carangiform swimming [60]. Such model was also compared against experimental data on golden shiners.

Specifically, undulations of the robotic fish and six additional golden shiner were analyzed using ProAnalyst (Xcitex Inc., Cambridge, MA, USA) motion tracking system. Technical details on the motion tracking analysis are presented in Figure S2, Figure S3, Figure S4, and Table S2 of Material S1. The comparison between model results and robotic fish undulations was subsequently performed to validate the ability of the robotic fish to reproduce carangiform swimming. Such trials were also used to measure the mean tail-beat frequency in live fish. Specifically, each fish was recorded with a high quality video camera (Canon Vixia HG 20) while swimming in the water tunnel at the speed of the water flow (the fish appeared at a stationary position in the water tunnel over time). The tail-beat frequency for golden shiners was measured as 3.32 Hz. Further details on the fish tail-beat frequency analysis are in Material S1.

**Figure 4:**
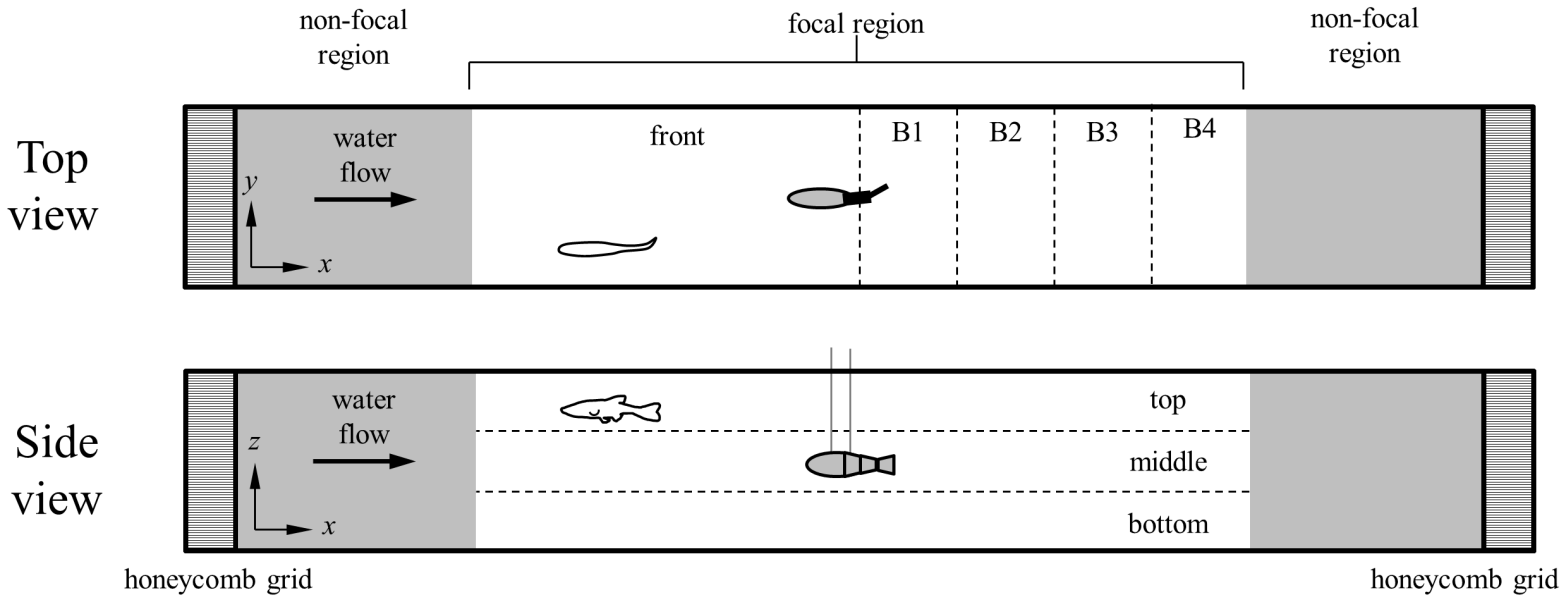
Top and Side views of the water tunnel with schematic illustration of the compartments forming the focal region. A live fish is considered to actively interact with the bioinspired robotic fish when the *x*-coordinate of its center of mass is in the focal region, which extends four body lengths from the *x*-coordinate of the robotic fish center of mass along its longitudinal axis. The focal region is partitioned into front, B1, B2, B3, and B4 compartments from the Top view. The same region is partitioned into top, middle, and bottom compartments from the Side view.

In the behavioral experiment, five minute long experimental trials were processed to discriminate the fish position from both the Top and Side views. Following [20], two main regions were defined for each perspective, by dividing the control volume into: i) the region of fish-robot interaction (referred to as the “focal region”), which extended eight fish body lengths and was centered on the robotic fish center of mass, and ii) the non-interaction zone, which consisted of the remaining portion and was delimited by the two plastic honeycombs, that is, the front-most and back-most regions, respectively, shown in Figure 4. In the Top view, the focal region was further divided in two equal sub-regions: i) frontal compartment (referred to as “front”) comprising the half of the focal region in front of the robotic fish and ii) four equal compartments of one body length each (referred to as “B1”, “B2”, “B3”, and “B4”, respectively) behind the robot, see Figure 4. Notably, the portion of the focal region behind the robotic fish identifies the microhabitat in which the flow perturbations induced by the robot beating tail are expected to generate relevant effects on golden shiners' swimming, as demonstrated in [20]. In the Side view, the focal region was divided in three compartments of equal depth: i) “top”, ii) “middle”, and iii) “bottom”, respectively, see Figure 4. The time spent by fish in each compartment was then recorded in all the experimental conditions from both perspectives. Data were averaged over the six repetitions for each experimental condition.

### Statistical analyses

Within the behavioral experiment, six trials were performed for each experimental condition and each trial was analyzed twice to compute the time spent by fish in each compartment, in both the Top and Side views. These numbers were initially resolved into a single value by considering the total time spent by each fish in the whole focal region (equal between the Top and Side views). Secondly, the total time spent by fish in each compartment was analyzed individually for the Top and Side views. Fish preference for a given condition was taken as proportional to the time spent in the vicinity of the bioinspired robotic fish and measured for the focal region, as well as for each compartment comprised in both the Top and Side views.

The color-effect induced by the robotic fish was ascertained using a paired t-test. The mean time spent by fish in the focal region of the test tank was compared between experiments with Gray robot and Red robot and the expected mean difference between paired observations was set to zero. In other words, the time spent by fish in the vicinity of the two prototypes was compared to detect the role of the robotic fish color pattern on fish spatial preference.

A one-way analysis of variance (ANOVA) was used to assess variations in the time spent by fish in the focal region or in each compartment of the focal region (front, B1, B2, B3, and B4 for the Top view and top, middle, and bottom for the Side view, respectively) among the experimental conditions. Specifically, for each experimental trial, the time spent in the focal region, or in each focal compartment, was the dependent variable and the condition was the independent variable. Notably, the time spent in the focal compartments of the test tank was analyzed in both the Top and Side views for both the prototypes, that is, two separate analyses were considered for the Gray robot and the Red robot, respectively, in relation to the Top and Side views.

A one-way ANOVA was also used to study variations in the time spent by fish in the part of the focal region where hydrodynamic advantage is most likely to occur, that is, behind the robotic fish and in the middle of the water column. Specifically, for each experimental trial, the time spent by fish swimming in the middle of the test tank and in compartments B1, B2, B3, and B4 was the dependent variable and the robotic fish color was the independent variable.

The tail-beat frequency was calculated for the six live fish by averaging the data from three distinct ten second long videos. The final value for the tail-beat frequency of live fish was calculated as the average of the six distinct main frequencies.

Data analysis was carried out using Statview 5.0. The significance level was set at p≤0.05. Fisher's protected least significant difference (PLSD) post-hoc tests were used where a significant main effect of the condition variable was observed.

## Results

**Figure 5:**
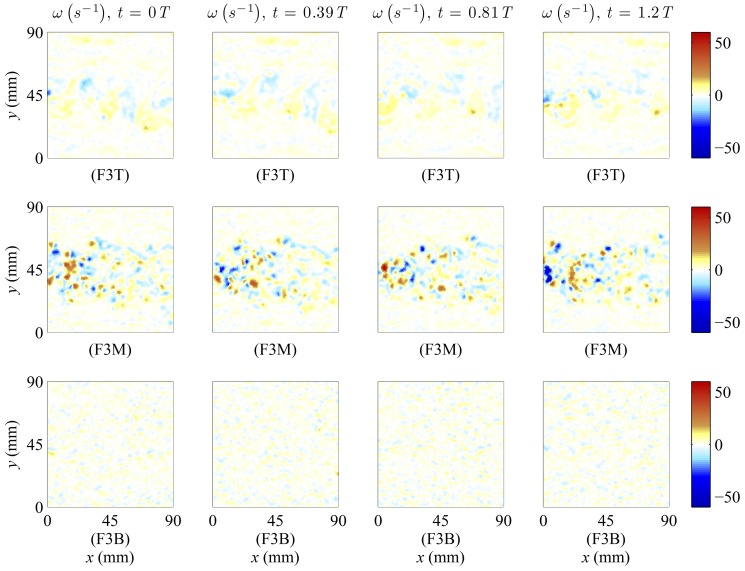
Time-resolved vorticity field measured in top (T), middle (M), and bottom (B) compartments of the Side view, respectively, with the robot tail-beat frequency set at 3 Hz. The time interval (*t*) between frames is scaled with respect to the tail-beat period (*T* = 0.33 seconds) corresponding to the tail-beat frequency of 3 Hz. The initial time (*t* = 0) does not correspond to the same tail configuration, as PIV analyses were independently executed for the three compartments. For the first row (T) the tail-beat amplitude is −12, 3, 0, and −3 mm, for the second row (M) it is −10, −1, 3, and −5 mm, and for the third row (B) it is −3, −1, 8, and −10 mm (measured as the lateral transverse displacement at the caudal fin terminal with respect to the *y*-axis). Red structures represent counterclockwise vortices and blue structures represent clockwise vortices. The regions of observation correspond to the three compartments defined for the Side view identified in Figure 4. (Online version in color).

The PIV experiment showed that the wake induced by the tail-beat of the robotic fish consists of a staggered array of trailing discrete vortices of alternating sign, as in [64], [65]. Specifically, the vortical structures shed in the flow are similar to the ‘double row reverse Karman street’ discussed in [60], [64]. This similarity was only observed in the middle compartment of the test tank behind the robotic fish, see Figure 5. For tail-beat frequencies of 2 Hz and 4 Hz, the time intervals between the generation of the vortices was lapse or compact, respectively, confirming that the shape and distribution of vortical structures in the robot's wake is controlled by its undulations. Conversely, the modest vorticity pattern in the top compartment was likely caused by the presence of the external rods [52], connected to the robot that act as bluff bodies, see Figure 5. Further information along with details on the PIV analysis is presented in Figure S5, Figure S6, and Figure S7 of Material S1.

**Figure 6:**
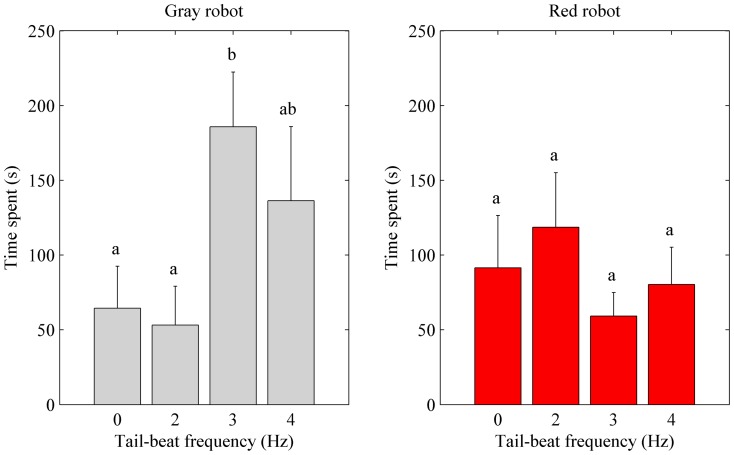
Behavioral experiment: mean time spent by fish within the focal region in each experimental condition (note that the total acquisition time was five minutes for each trial). Gray histograms represent the mean time spent in the vicinity of the Gray robot and red histograms represent the mean time spent in the vicinity of the Red robot. Means not sharing a common superscript are significantly different (Fisher's PLSD, p<0.05). (Online version in color).

**Figure 7:**
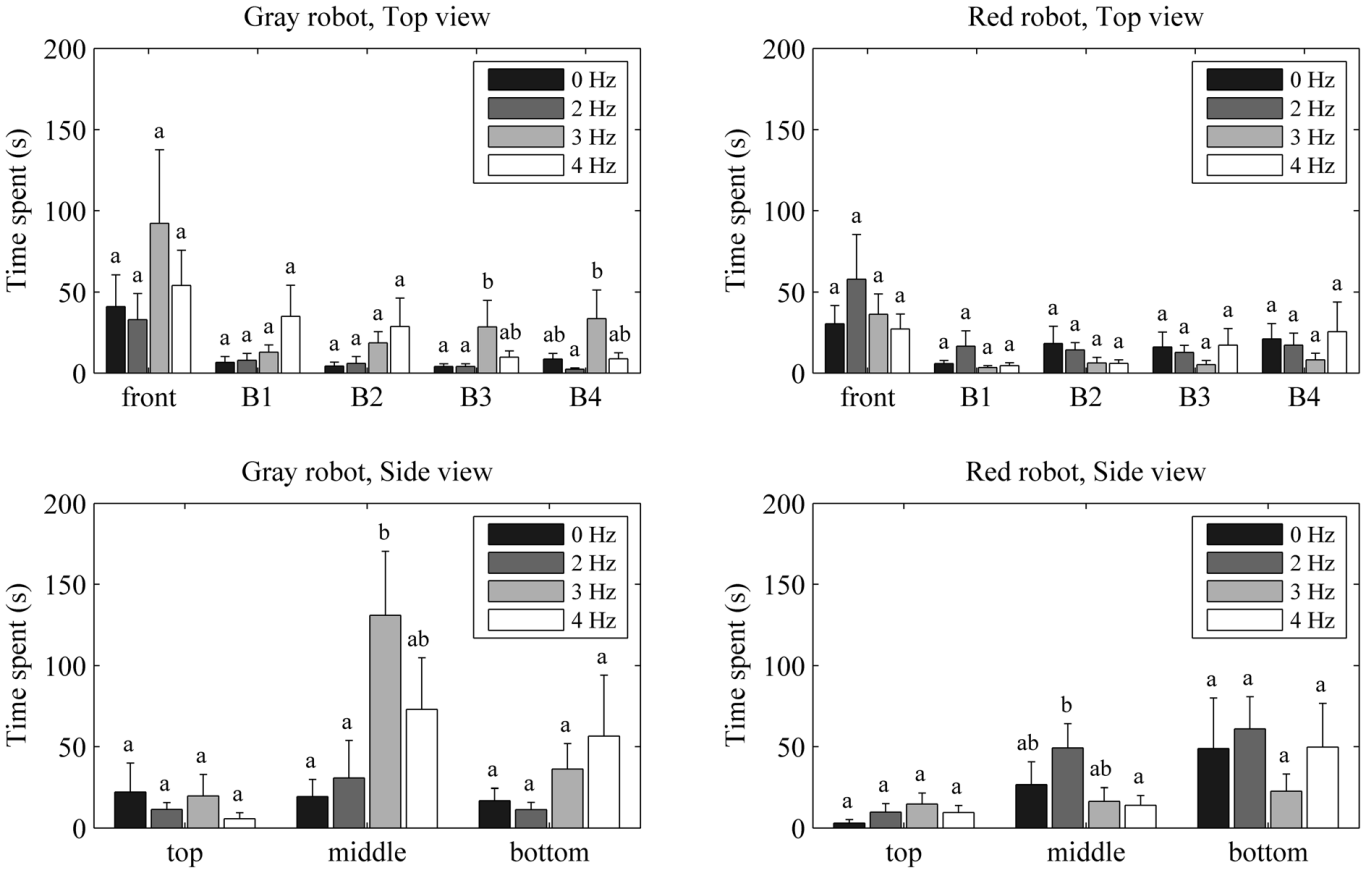
Behavioral experiment: mean time spent by fish in each compartment of the focal region across the experimental conditions (note that the total acquisition time was five minutes for each trial). Histograms represent the mean time spent in the vicinity of the Gray robot (left) and the Red robot (right) with respect to both Top view (top) and Side view (bottom). Error bars refer to the standard error. Means not sharing a common superscript are significantly different (Fisher's PLSD, p<0.05).

In the behavioral experiment, an influence of the robotic fish color pattern on the time spent by live fish in the focal area was not detected (mean difference: 22.6 s, p = 0.38), see Figure 6. However, fish spent significantly more time in the vicinity of the Gray robot over the Red robot when its tail-beat frequency was set to 3 Hz (mean difference: 126.6 s, p ≤ 0.05). In other words, for a tail-beat frequency matching the spontaneous tail-beat frequency of live fish, the naturally colored robotic fish was more attractive than the red replica. Furthermore, the influence of the robot tail-beat frequency on fish positional preference varied according to the robot color pattern. Specifically, when considering the Gray robot, the time spent by fish in each compartment of the focal region was a function of the robot tail-beat frequency, while no significant variations were observed in the Red robot, see Figure 7.

### Gray robot

A significant difference in the time spent by fish in the focal region was observed across the experimental conditions (F_3, 20_ = 2.97, p≤0.05), see Figure 6. Namely, the time spent in the focal region was affected by the Gray robot tail-beat frequency. Furthermore, post-hoc comparisons revealed that fish spent significantly more time in the vicinity of the bioinspired robotic fish as its tail-beat frequency was set to 3 Hz (185.8 s) as compared to the case when the robot tail-beat frequency was 0 Hz or 2 Hz (121.3 s or 132.7 s, respectively). The time spent in the non-focal region of the test tank complemented the time spent in the focal region, that is, the mean time spent within the non-focal region was the lowest for a tail-beat frequency of 3 Hz (114.2 s).

#### 1. Top view

The time spent by fish in the front compartment was not affected by the tail-beat frequency of the Gray robot, that is, the time spent in the front compartment did not significantly vary across experimental conditions. Similarly, fish did not show significant differences in the time spent in compartments B1, B2, B3, and B4 across the experimental conditions. The time spent in compartment B3 (28.5 s) was the highest when the robot tail-beat frequency was set to 3 Hz, and post-hoc comparisons revealed that such time was significantly different than the time spent in B3 for the robot beating its tail at 0 Hz or 2 Hz (24.5 s or 24.6 s, respectively), see Figure 7. Fish also showed the highest amount of time spent in compartment B4 when the robotic fish tail-beat frequency was set to 3 Hz (33.6 s), and post-hoc comparisons revealed that this time was significantly different than the time spent in B4 for the robot beating its tail at 2 Hz (31.2 s), see Figure 7.

#### 2. Side view

The time spent by fish in the top compartment was not affected by the tail-beat frequency of the Gray robot, that is, the time spent in the top compartment did not significantly vary across the experimental conditions. Conversely, a significant difference was observed in the time spent by fish in the middle compartment across the experimental conditions (F_3, 20_ = 3.20, p≤0.05). The time spent in the middle compartment (131.0 s) was maximum when the robot tail-beat frequency was set to 3 Hz, and post-hoc comparisons indicated that such time was significantly different than the time spent in the middle compartment for the robot beating its tail at 0 Hz and 2 Hz (111.7 s and 100.4 s, respectively), see Figure 7. Similarly to the analysis for the top compartment, the time spent by fish in the bottom compartment was not affected by the tail-beat frequency of the robot, see Figure 7.

### Red robot

A significant difference in the time spent in both focal and non-focal regions was not observed across the experimental conditions, see Figure 6. In other words, the time spent in both regions was not affected by the Red robot tail-beat frequency. Moreover, post-hoc comparisons did not reveal significant differences in the time spent by fish in the two regions between experimental conditions.

#### 1. Top view

The time spent by fish in the front compartment was not affected by the tail-beat frequency of the Red robot. Namely, the time spent in the front compartment did not significantly vary across experimental conditions. Similarly, fish did not show significant differences in the time spent in compartments B1, B2, B3, and B4 across the experimental conditions, see Figure 7.

#### 2. Side view

The time spent by fish in the top, middle, and bottom compartments was not affected by the tail-beat frequency of the Red robot. In other words, the time spent in each of these compartments did not significantly vary across the experimental conditions. The time spent by fish in the middle compartment (49.2 s) was the highest when the robot tail-beat frequency was set at 2 Hz and post-hoc comparisons revealed that such time was significantly different than the time spent in the middle compartment for the robot beating its tail at 4 Hz (35.2 s), see Figure 7.

### Detailed analysis of spatial preference behind the robotic fish

The time spent by fish in compartments B1, B2, B3, and B4 (Top view), while swimming in the middle compartment of the water column (Side view), was affected by the coloration of the robot when beating its tail at 3 Hz (F_1, 46_ = 5.08, p ≤ 0.05), data not shown. Table 1 reports the percentage of the time spent by fish swimming in the middle compartment of the water column in each of the four regions of interest behind the robotic fish, with respect to the total mean time spent within the whole focal region when the robots beat their tails at 3 Hz.

**Table 1:** Mean time spent by fish in each of the four one-body length compartments behind the robot (Top view) while in the middle compartment of the water column (Side view).

	B1	B2	B3	B4
Gray robot	15.1	3.4	7.0	6.9
Red robot	0.7	3.6	4.7	3.8

Data refer to the percentage of time with respect to the mean time spent in the whole focal region for the conditions with the robots beating their tail at 3 Hz.

## Discussion

Our results show that fish positional preference is affected by the color of the robotic fish, whereby a prototype with a bioinspired color pattern (Gray robot) is more attractive than a red replica (Red robot). This result is in line with experimental evidences on the role of visual cues in computer-animated images to elicit social responses in comparable fish species, such as sticklebacks [66], [67], mosquitofish [51], and zebrafish [47], [48], . Specifically, several phenotypic varieties of zebrafish, taxonomically listed in the *Cyprinidae* family together with golden shiner, are known to react differently to computer animations of their conspecifics depending on the similarities of their stripe pattern [47], [48] and color pigmentation [48]. Further studies have corroborated the evidence that zebrafish shoaling preference is affected by visual cues incorporated by both live [72] and robotic [35], [37], [73] stimuli.

While the evolution of the stripe patterns is generally attributed to ecologic constraints and is associated with structurally complex habitats, the evolution of color patterns, not due camouflage or other ecologic constraints, is commonly related to fish mating choice [74]. Red phenotypic variants of golden shiners are not present in nature and other red-colored species do not co-inhabit water bodies where golden shiner is native [61]. Zebrafish ecology is, in this regard, similar to golden shiners, whereby red phenotypes do not exist and interaction with red-colored species is not documented [75]. Laboratory studies have demonstrated that shoaling preference in zebrafish is negatively affected by red pigmentations of animated images of their conspecifics [48], which are likely perceived as heterospecifics [48]. In line with [48], we observe that fish preference is significantly higher when the species-specific color pattern is experimentally integrated into the robotic prototype.

The spatial preference of live fish in the test tank suggests that, beyond the visual cues offered by the robot, flow cues play an important role in shaping fish-robot interactions. The attraction induced by the robot tail-beat on fish is already known in the literature [20], [35]. Here, the robotic fish was designed to mimic the locomotion of golden shiners and match their morphology. The flow physics induced by the robotic fish tail-beat was measured with PIV and juxtaposed with the spatial preference of live fish to dissect the role of flow cues on the interaction. Despite the large amount of time spent by fish outside the focal region, we observe that the time spent by subjects in both the middle compartment (from the Side view) and behind the robot (from the Top view) were the highest when the Gray robot matched the tail-beat frequency of the live fish. Specifically, fish consistently preferred to follow the Gray robot rather than its red replica, spending a larger amount of time in the focal region behind the robotic fish (from the Top view) and in the middle of the water column (from the Side view). In other words, fish preferred to spend time following the Gray robot when its undulation matched their locomotion pattern at that flow speed.

We hypothesize that such preference is due to the wake produced by the tail-beat of the robotic fish, which seeks to replicate the flow physics induced by the motion of a conspecific. The latter feature is addressed through the design of a miniature multi-link mechanism that allows for replicating the species-specific locomotory pattern of carangiform swimmers [64], wherein a large portion of the body undulates to propel the animal. Such interaction is likely to produce a hydrodynamic advantage for the live fish, which thus would follow the Gray robot to reduce its energy expenditure, in agreement with observations on other social carangiform swimmers [64]. The interpretation that fish preference for the robot is modulated by flow cues is supported by evidence in [20], where it is demonstrated that golden shiners tend to follow a larger robotic fish, whose morphology is not directly inspired by golden shiners, on the basis of its hydrodynamics. However, while hydrodynamic advantage is known to be a primary determinant of shoaling within conspecifics and heterospecifics [76], our results indicate that golden shiners respond differentially to the same flow cues induced by a morphophysiologically-inspired robotic fish as a function of its coloration.

The study of robot-animal interaction is an interdisciplinary research field known as “ethorobotics” that is receiving increasing interest by both the engineering and biology communities. In this emerging context, robots can be designed to offer controlled stimuli in laboratory experiments toward dissecting salient behavioral responses. Robotic fish that incorporate biologically relevant attributes of live animals have been shown to influence fish behavior across a wide spectrum of sensory modalities [20], [22], . In this study, we have proposed an implementation of a robotic fish to investigate the interplay between visual and flow cues in the phenomenon of schooling in carangiform social fish.

## Acknowledgments

The authors would like to gratefully acknowledge Dr. A. L. Facci for the technical support on PIV measurements, Mr. M. Drago for the technical support on the design of the robotic fish prototype, Ms. L. Yang and Mr. K. Khan for their valuable help in performing the experiments, and Dr. S. Macri for the useful discussion and the assistance with the behavioral classification using the software Observer. The authors would also like to acknowledge the Wildlife Conservation Society's Department of Herpetology at the Bronx Zoo for providing the experimental animals.

## Supporting Information

Material S1 **Supporting material on the visual aspect of the robotic fish, the measurement of golden shiners' tail-beat frequency, the motion tracking of the robotic fish, the particle image velocimetry analyses.**
(DOCX)
