# Supplementary material for: Fish and Robots Swimming Together in a Water Tunnel: Robot Color and Tail-Beat Frequency Influence Fish Behavior
Source: PLoS One. 2013 Oct 25;8(10):e77589. doi: 10.1371/journal.pone.0077589 (PMC3808421; doi:10.1371/journal.pone.0077589)
Supplement: Material S1 — Supporting material on the visual aspect of the robotic fish, the measurement of golden shiners' tail-beat frequency, the motion tracking of the robotic fish, the particle image velocimetry analyses. (DOCX) [file pone.0077589.s001.docx]

**MATERIAL S1**

**ROBOTIC FISH VISUAL ASPECT**

The two robotic fish prototypes were painted to mimic the natural golden shiners’ colour pattern or were coloured in red, respectively, according to the description provided in the main manuscript, see Figure S1.


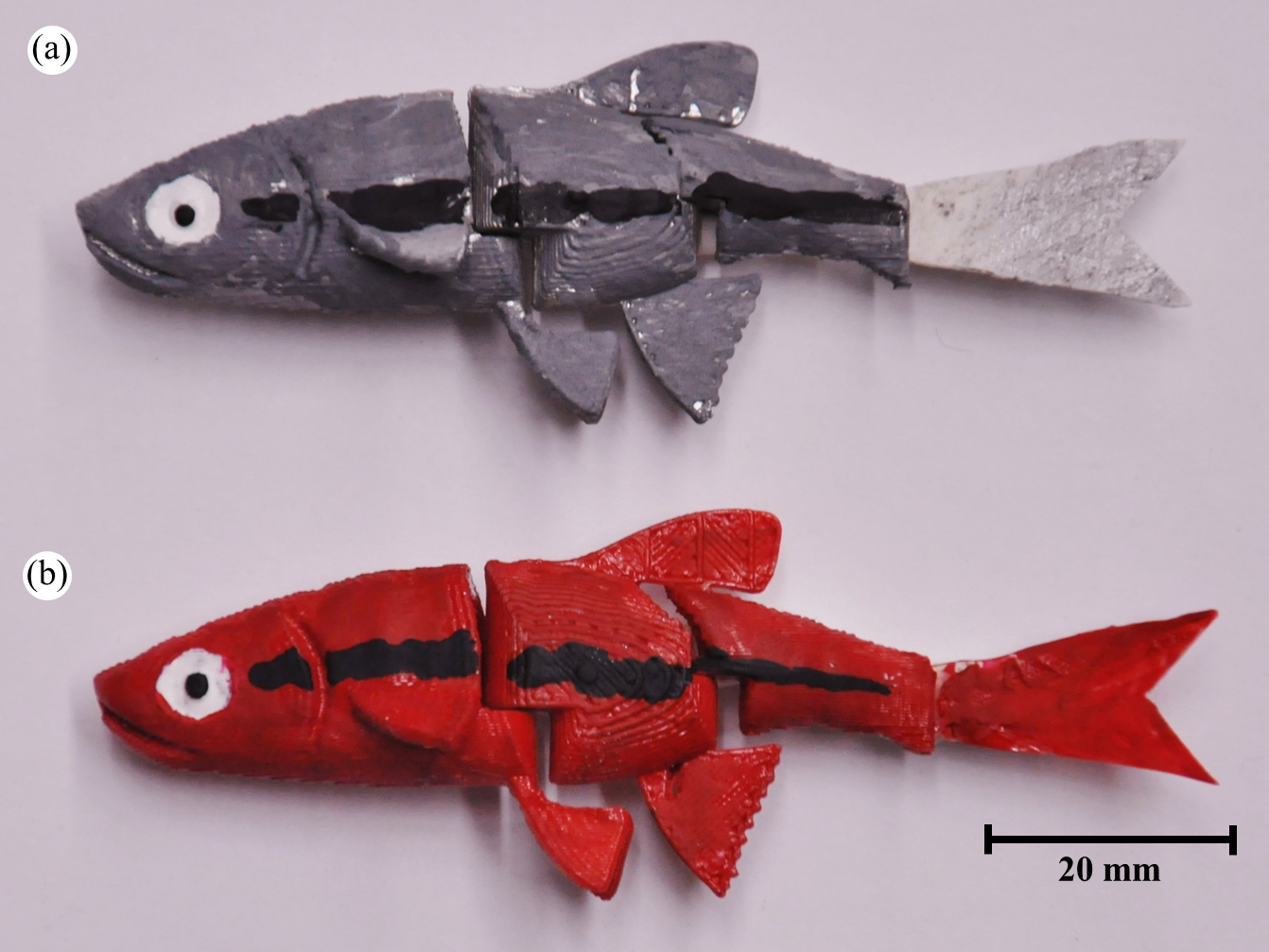


**Figure S1.** Picture of the two bioinspired robotic fish used in the experiment: (a) Gray robot and (b) Red robot.

Table S1 reports the proportions used in the design of the robotic fish.

|  | Length (cm) |
| --- | --- |
| Pectoral fin | 1.02 |
| Dorsal fin | 1.71 |
| Pelvic fin | 1.21 |
| Anal fin | 1.00 |
| Caudal fin | 2.19 |

**Table S1**. Relevant dimensions of parts of the robotic fish.

**TAIL-BEAT FREQUENCY OF GOLDEN SHINERS**

Six naïve golden shiners (*Notemigonus crysoleucas*) were used to determine the average fish tail-beat frequency when swimming in the test tank at the constant speed of ten cm/s. Fish were recorded with a video camera (Canon Vixia HG 20) for five minutes each. Three videos, each ten seconds long, were selected out of the observation period and used to calculate the average tail-beat frequency for each fish. The videos were analysed frame by frame to measure the number of tail-beat periods in the time interval. This procedure was repeated for all the subjects and then averaged. The mean tail-beat frequency for golden shiners was 3.32 Hz, with a standard error of 0.23 Hz.

**MOTION TRACKING OF THE ROBOTIC FISH**

To assess the similarities between the undulations of the robotic fish and the live subjects, the motions of the robotic fish, for different tail-beat frequencies of 2 Hz, 3 Hz, and 4 Hz, and a single live subject were measured using the software ProAnalyst (Xcitex Inc., Cambridge, MA, USA). Five points along the longitudinal length of the robotic fish body were tracked, see Figure S2. The points tracked on the live subject were selected to maintain the same proportions as Figure S2.


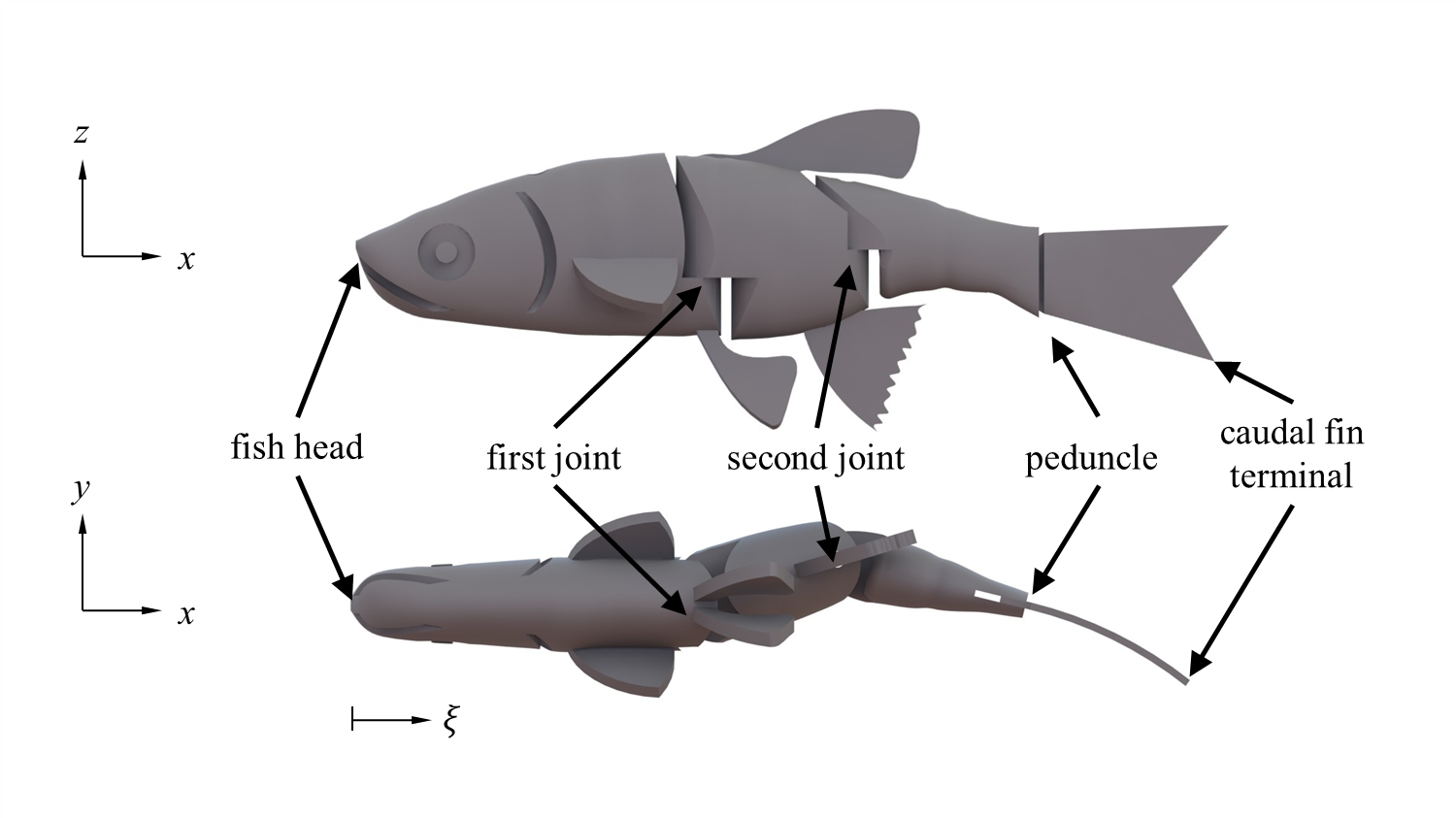


**Figure S2.** Tracked points along the robotic fish longitudinal length.

For comparison, each undulation was fitted using the classical Lighthill’s model of carangiform swimming, which consider a parabolic envelope for the undulation and a monochromatic propulsive wake [[1](#_ENREF_1)]. Specifically, the model transverse displacement *η* at the abscissa *ξ* and time *t* is

(1)

where *k* is the wave number and *f* is the tail-beat frequency.

The fitting of the model parameters was performed in MATLAB (The MathWorks Inc., Natick, Massachusetts, USA) by minimizing the error defined by

(2)

where *ξ_i_* with *i* = 1, ..., *M* = 5 are the abscissas of the tracked points, *t_j_*, *j* = 1, ..., *N*, are the sampling instants, and *η^exp^* identifies experimental data. Such error is scaled between 0 and 1, with null error representing perfect match between model results and experiment and unit error identifying complete discrepancy.

Table S2 demonstrates the model accuracy in describing the locomotion of golden shiners (*E* = 0.096), which, in turn, offers evidence for the ability of the robotic fish to replicate golden shiners’ locomotion (*E* ≤ 0.330).

|  | *c*_1_ | *c*_2_  (cm^-1^) | *k*  (rad cm^-1^) | *E* |
| --- | --- | --- | --- | --- |
| Robotic fish, *f* = 2 Hz | -0.056 | -0.017 | 0.66 | 0.321 |
| Robotic fish, *f* = 3 Hz | 0.021 | -0.034 | 0.67 | 0.259 |
| Robotic fish, *f* = 4 Hz | -0.112 | 0.074 | 0.53 | 0.330 |
| Golden shiner, *f* = 3.72 Hz | -0.073 | 0.057 | 0.51 | 0.096 |

**Table S2.** Identified parameters of the model of carangiform swimming.

Figure S3 and Figure S4 display the undulations of the robotic fish beating its tail at 3 Hz and the golden shiner, respectively.

**Figure S3.** Comparison of model results and experimental data for the robotic fish beating its tail at 3 Hz.

**Figure S4.** Comparison of model results and experimental data for the golden shiner.

**PARTICLE IMAGE VELOCIMETRY MEASURES**

The particle image velocimetry (PIV) technique [[2](#_ENREF_2),[3](#_ENREF_3)] was adopted to study the flow physics in the vicinity of the robotic fish. The PIV experiment was arranged as described in the experimental setup and protocol section of the main manuscript. A high speed camera was positioned underneath the test tank to collect images within a 90x90 mm^2^ frame at an exposure of 2.5 ms. The captured PIV images pairs were processed using adaptive correlations [[3](#_ENREF_3),[4](#_ENREF_4)] into velocity and vorticity fields generated in FlowManager (Dantec Dynamics Inc., Denmark) and graphically displayed using MATLAB. The height of the water level in the test tank was constantly maintained at 14 cm and divided in three equal compartments from the laser perspective. The PIV measurements were performed at 2.33 cm (bottom compartment), 7.00 cm (middle compartment), and 11.67 cm (top compartment), respectively, from the bottom surface of the test tank. A total of twelve PIV measurements were performed as the tail-beat frequency of the robotic fish and the illumination plane were varied, see Figure S5.

**Figure S5.** Illumination planes for the PIV study along with the presentation of the three compartments in the focal region.

The vorticity field was extracted by processing the velocity data [[2](#_ENREF_2)], Figure S6.

**Figure S6.** Vorticity profiles centred in each of the three compartments, top (T), middle (M), and bottom (B) with the robot tail-beat frequency set at 2 Hz, 3 Hz, and 4 Hz. For the first row the tail-beat amplitude is 0, -6, -8, and 5 mm, for the second row it is 0, 10, 2, and -11 mm, and for the third row it is 0, 0, -5, and -9 mm (measured as the transverse displacement at the caudal fin terminal with respect to the *y* axis). (Online version in colour).

The vorticity pattern observed in the middle compartment of the focal region resembles a ‘double row reverse Karman street’ [[4](#_ENREF_4),[5](#_ENREF_5)] and the vortex shedding is controlled by the tail-beat frequency [[6](#_ENREF_6)]. The vortex pairs alternated symmetrically behind the robotic fish in a typical ‘V’ pattern while drifting downstream with the water flow, see Figure S7.

**Figure S7.** Drift of a vortex pair for the tail-beat frequency of the robotic fish set to 3 Hz. The circle identifies a vortex pair. The time interval (*t*) between frames is scaled with respect to the tail-beat period (*T* = 0.33 seconds) corresponding to the tail-beat frequency of 3 Hz. The tail-beat amplitude is -10, 0, 3, and -5 mm (measured as the transverse displacement at the caudal fin terminal with respect to the *y* axis). (Online version in colour).

In the bottom compartment, no significant vorticity is evidenced for each of the robotic fish tail-beat frequencies, see Figure S6. Conversely, in the top compartment, considerable vorticity was induced by the Plexiglas rods, as made clear by the comparison between vorticity fields for tail-beat frequency of 0 Hz and 3 Hz, see Figure S6. Indeed, the vorticity induced by the rods did not qualitatively vary as the robot’s tail-beat frequencies was changed, see Figure S6. Conversely, the vorticity profiles varied significantly within the middle compartment as the robot tail-beat frequency changed from 0 Hz to 3 Hz, see Figure S6.

**REFERENCES**

1. Lighthill J (1987) Mathematical Biofluiddynamics. Cambridge: Society for Industrial and Applied Mathematics.

2. Raffel M, Willert C, Kompenhans J (1998) Particle image velocimetry: A practical guide. New York: Springer.

3. Scarano F, Riethmuller ML (1999) Iterative multigrid approach in PIV image processing with discrete window offset. Exp Fluids 26: 513-523.

4. Sfakiotakis M, Lane DM, Davies JBC (1999) Review of fish swimming modes for aquatic locomotion. IEEE J Oceanic Eng 24: 237-252.

5. Borazjani I, Sotiropoulos F (2008) Numerical investigation of the hydrodynamics of carangiform swimming in the transitional and inertial flow regimes. J Exp Biol 211: 1541-1558.

6. Epps BP, Valdivia y Alvarado P, Youcef-Toumi K, Techet AH (2009) Swimming performance of a biomimetic compliant fish-like robot. Exp Fluids 47: 927-939.
